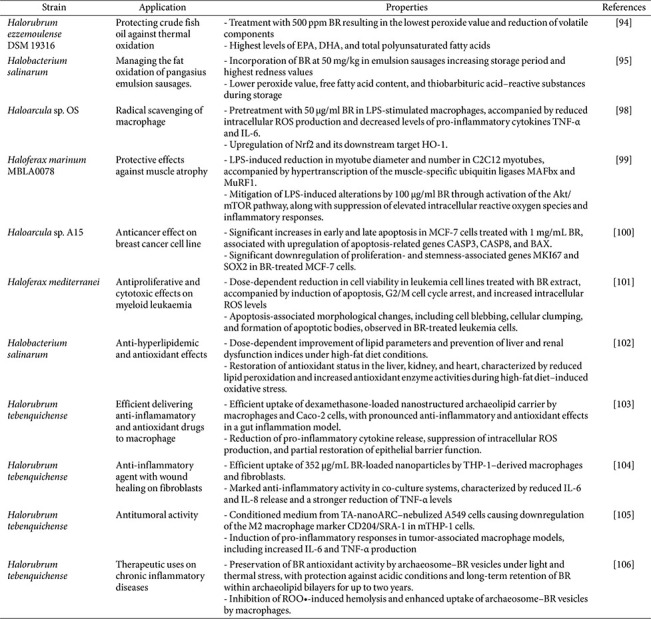# Recent Advances in Understanding Bio-Compounds from Haloarchaea

**DOI:** 10.4014/jmb.2601.01034

**Published:** 2026-02-24

**Authors:** Chi Young Hwang, Myung-Ji Seo

**Affiliations:** 1 Department of Bioengineering and Nano-Bioengineering, Incheon National University, Incheon 22012, Republic of Korea; 2 Division of Bioengineering, Incheon National University, Incheon 22012, Republic of Korea; 3 Research Center for Bio Materials & Process Development, Incheon National University, Incheon 22012, Republic of Korea

**Keywords:** Haloarchaea, Enzymes, Polyhydroxyalkanoates, Bacterioruberin, Biotechnology

## Abstract

Haloarchaea are extreme halophilic microorganisms that thrive in hypersaline environments. They possess unique physiological, metabolic, and molecular adaptations that enable robust biomanufacturing under harsh conditions. Growing taxonomic diversity, advances in genome sequencing, and the development of genetic and process engineering tools have significantly expanded interest in haloarchaea as non-conventional microbial cell factories. This review summarizes recent advances in the biosynthesis of high-value bio-compounds and their properties by haloarchaea. Particular emphasis is placed on industrially relevant enzymes, biodegradable polyhydroxyalkanoates, and the functional carotenoid bacterioruberin. Our investigation provides comprehensive information on the strategies, mass production, properties, and applications for the production of these bio-compounds from halophilic archaea. This review suggests the potential of haloarchaea in the industry and biotechnology as cellular factories to produce diverse bioactive compounds.

## Introduction

**Fig. 1:**
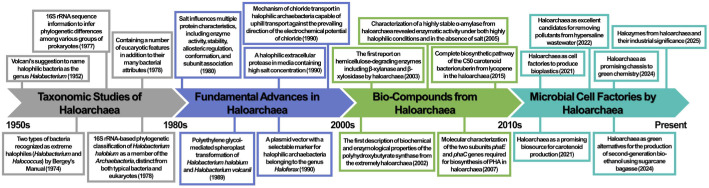
Timeline of key advancements in haloarchaea research. Each time point summarizes representative research of haloarchaea, including taxonomic studies, fundamental advances, bio-compound production, and development as microbial cell factories.

Haloarchaea inhabit a wide range of high-salt environments (7~35% w/v NaCl), including saline lakes, solar salterns, and salted or fermented food products [1-3]. These microorganisms possess specialized physiological and molecular adaptations that enable them to survive and proliferate under extreme osmotic stress [4]. In 1977, Woese and Fox used 16S rRNA sequence data to infer phylogenetic relationships among diverse prokaryotic groups [5]. Their analysis revealed that a distinct lineage of aerobic, often red-pigmented extreme halophiles and thermophiles belonged to a newly defined domain Archaea. The best-known Haloarchaea belong to the archaeal phylum *Methanobacteriota*, reclassified in 2023 [6]. Taxonomic diversity within the class *Halobacteria*, which comprises the most prominent halophiles belonging to the phylum *Methanobacteriota*, has expanded substantially in recent years. As of December 2025, the class includes 8 families, 86 genera, and 457 validly published species, representing an increase of 100 species since December 2023. Compared with the December 2023 census, which reported 357 species across 82 genera and 9 families with validly published names, the current classification reflects nearly a twofold increase relative to the May 2017 survey, which documented only 233 species in 57 genera and 6 families [7-9]. This steady expansion in the isolation and phylogenetic reclassification of halophilic archaeal species reflects both the increasing recognition of their genetic diversity and the growing availability of genome sequencing data [10]. It also continuously demonstrates the rising scientific interest in haloarchaea and the intensified research efforts directed toward these microorganisms (Fig. 1).

Their mechanisms for surviving in high-salt environments have also driven the natural production of a wide variety of unique biomolecules. Haloarchaea achieve this in part through the regulation of intracellular and extracellular ionic balance and the production of compatible solutes [11]. They generate energy through diverse metabolic pathways, including aerobic and anaerobic respiration and fermentation, and can also harvest light energy for ATP synthesis via bacteriorhodopsin [12, 13]. Their characteristic red to pink pigmentation arises from carotenoids, which not only contribute to their distinctive coloration but also protect cells against UV radiation–induced damage [14]. Additional survival mechanisms include specialized transport systems and unique cell membranes composed of ether-linked lipids [15]. In hypersaline habitats, halophilic archaea rely on Na^+^ ions to stabilize cellular structures such as membranes, ribosomes, and enzymes [16]. Unlike most non-halophilic bacteria, haloarchaea possess cell envelopes dominated by extensively glycosylated S-layers rich in negatively charged saccharides [17]. Their intracellular protein production machinery is adapted to high ionic strength, preventing protein aggregation and maintaining enzymatic activity [18, 19]. Haloarchaeal enzymes typically feature highly acidic, polar surfaces compatible with the surrounding ionic environment [11, 20]. Cytoplasmic proteins contain an unusually high proportion of acidic amino acids, particularly aspartate residues, whose negative charges are shielded by abundant Na^+^ ions. This property is an essential feature for preserving cellular integrity in extreme salinity [21].

**Fig. 2:**
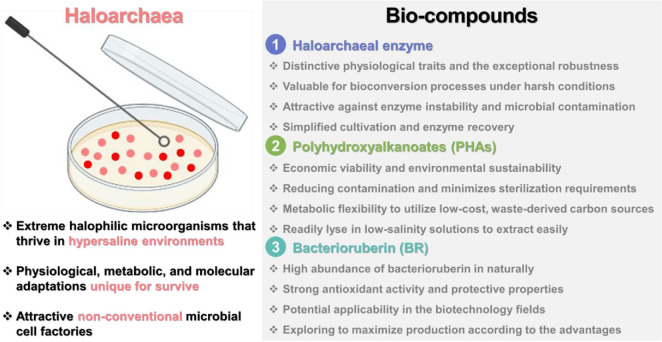
Bio-compounds from haloarchaea focused on this review.

In summary, haloarchaea display diverse metabolic capabilities and biological activities that make them highly attractive for biotechnological applications. This review focuses on recent advances in understanding haloarchaea for biosynthesis of valuable bio-compounds and their properties, particularly enzymes, biodegradable plastics, and functional materials for food and pharmaceutical applications (Fig. 2).

## Extreme Haloarchaeal Enzymes for Bioconversion

The distinctive physiological traits of haloarchaea and the exceptional robustness of their enzymes make them valuable biocatalysts for bioconversion processes operating under harsh industrial conditions [22]. Haloarchaeal enzymes are particularly attractive for industries challenged by enzyme instability and microbial contamination, as their intrinsic salt dependence enhances operational stability and process robustness. Moreover, haloarchaea offer practical advantages for industrial applications, including simplified cultivation and enzyme recovery. Their requirement for high salinity naturally suppresses contaminating microorganisms, enabling cost-effective downstream processing (Table 1) [23].

## Cellulase

Genomic and functional studies have demonstrated that haloarchaea harbor a wide range of cellulase and hemicellulase genes [24-27]. Several haloarchaeal cellulases and xylanases have been experimentally validated and shown to depolymerize plant biomass under high-salt conditions that inhibit conventional enzymes [27, 28]. For example, the extremely halophilic archaeon *Halorhabdus utahensis* produces β-xylanase and β-xylosidase enzymes that remain active at 5–15% and 5% (w/v) NaCl, respectively, and exhibit moderately thermophilic properties with optimal activity detectable up to 70°C [28]. Such haloarchaeal xylanases are promising for industrial bioconversions, including pentose sugar release for bioethanol production, paper pulp bleaching, fruit juice clarification, and improvement of animal feed digestibility. Cellulases from *Haloarcula* and *Haloferax* species exhibit optimal activity at 3.0–3.5 M NaCl, whereas those from *Natronobiforma cellulositropha* and *Hrd. utahensis* show maximal activity at higher salinities (4.0–5.0 M NaCl) under slightly alkaline conditions [24, 25, 29, 30].

## Protease

Haloarchaeal proteases, commonly classified as serine proteases (halolysins), exhibit remarkable resistance to extreme environmental conditions, including high salinity, temperature, and pH [31]. These properties align with the increasing industrial demand for robust proteases capable of maintaining activity under harsh processing conditions [32, 33]. The first haloarchaeal hydrolase to be purified and characterized was a serine protease from *Halobacterium salinarum*, which exhibited catalytic activity only at NaCl concentrations exceeding 2 M [34]. Consistent with this observation, most haloarchaeal proteases display optimal activity at high salt concentrations. *Hfx. lucentensis* strain GUBF-2 (MG076078) exhibited the highest protease activity, reaching 101.9 U/mL under 30% NaCl conditions, while *Halorubrum ezzemoulense* retained proteolytic activity across a NaCl range of 10–25% [35, 36].

More recently, increasing attention has focused on haloarchaeal proteases that retain activity under low-salt conditions. A protease from *Halogranum rubrum* demonstrated high hydrolytic activity toward myosin over a broad salinity range of 0–3 M NaCl [37]. Similarly, the HlyB protease from *Haloarchaeobius* sp. FL176 exhibited maximal activity at 0.5 M NaCl and retained 96.4% of its azocasein-degrading activity even at a low salt concentration of 0.37 M [38]. In addition, several *Halococcus* species produce proteases with broad salt tolerance. The extracellular protease HlyA from *Hcc. salifodinae* maintained more than 75% of its maximum activity across a NaCl range of 0.5–4 M [39, 40], while halolysin HlyHap from the same species exhibited optimal activity at 0.25–0.5 M NaCl under moderate temperature and slightly alkaline conditions [41]. These previous results suggest the potential of haloarchaeal proteases as low-salt-adapted biocatalysts for diverse industrial applications, including salt-fermented food processing [40].

## Amylase

The capacity of haloarchaea to produce and secrete hydrolytic enzymes, including amylases involved in the degradation of extracellular polysaccharides, has also been well documented [42]. Haloarchaea produce diverse amylolytic enzymes, such as α-amylases, glucoamylases, pullulanases, and amylopullulanases [43-45]. An amylase from the novel halophilic archaea *Natronococcus amylolyticus* Ah-36, isolated from Lake Magadi (a soda lake) in Kenya, was purified and reported for the first time [46]. Haloarchaeal amylases are typically highly stable under high-salt conditions and often retain activity in alkaline environments [47]. For example, the extracellular amylase AmyA from *Haloterrigena turkmenica* produced various sugar oligomers from starch, exhibiting optimal activity at 55°C, pH 8.5, and 2 M NaCl [48]. In contrast, the amylase from *Halococcus* sp. GUVSC8 hydrolyzed starch into glucose and maltooligosaccharides at 55°C, pH 6.0, and 2 M NaCl [49]. *Haloarcula* sp. HS produces both intracellular and extracellular amylases, with optimal activities at 60°C and 25% NaCl, reaching 120 U/mg and 350 U/mg, respectively [50].

The high catalytic performance of haloarchaeal amylases under saline conditions suggests their suitability for applications in agriculture and food processing, including wastewater treatment and bakery waste valorization, where conventional enzymes are often inhibited [48, 50]. For industrial application of haloenzymes, production of amylase from *Hfx. mucosum* MS1.4 was optimized to approximately 949.46 U/min through statistical optimization of incubation time, starch concentration, and NaCl concentration using response surface methodology (RSM) [51]. Nevertheless, compared with proteases, studies on the large-scale production of haloarchaeal amylases remain limited, underscoring the need for continued research to enable their efficient industrial deployment.

## Bioplastic and Biopolymer Biosynthesis

Developing biodegradable plastics with economic viability and environmental sustainability has become a global strategy for addressing the challenges, such as environmental pollution, dependence on fossil resources, and the persistence of conventional plastics [52]. Among various microorganisms, haloarchaea are attractive microbial cell factories for biodegradable polymers, particularly polyhydroxyalkanoates (PHAs) [23, 53]. In 1986, the presence of polyhydroxybutyrate (PHB) was reported in four haloarchaeal species, *Hfx. mediterranei*, *Hfx. volcanii*, *Hfx. gibbonsii*, and *Har. hispanica* [54]. Haloarchaea can be cultivated at extremely high salt concentrations, which substantially reduces contamination risks and minimizes sterilization requirements, thereby lowering overall fermentation costs [55]. Their metabolic flexibility allows them to utilize low-cost, waste-derived carbon sources such as whey, wastewater, and refined glycerol [56]. Many haloarchaeal species, including *Hfx. mediterranei*, can assimilate a wide range of organic substrates and channel excess carbon into PHA biosynthesis under nutrient-limited conditions [57]. The combination of high salt tolerance and the ability to valorize diverse waste substrates positions haloarchaea as a practical and economically attractive platform for large-scale PHA production [55, 57]. Furthermore, haloarchaeal cells readily lyse in low-salinity solutions such as water. This characteristic offers an economical and environmentally friendly solution to downstream processing, which constitutes a major cost component in PHA production (Table 2).

## Cost-Effective PHA Production Using Non-Conventional Sources

The most extensively studied PHA-producing haloarchaeon biosynthesizes poly(3-hydroxybutyrate-co-3-hydroxyvalerate) (PHBV), less crystalline and more flexible, comprising a copolymer with 3-hydroxybutyrate (3HB) and 3-hydroxyvalerate (3HV) [58, 59]. Research focused on the sustainable, large-scale production of PHBV using non-conventional carbon sources is particularly attractive. Among them, starch is an attractive carbon source for producing PHA by halophilic archaea. When starch was used as the low-cost carbon source, *Hrr. chaoviator* CEJ34-14, *Natronomonas pallidum* CEJ5-14, and *Har. tradensis* CEJ48-10 were identified as the most effective PHA producers, accumulating 9.25%, 7.11%, and 1.42% of their DCW, respectively [60]. Culture-optimized *Haloarcula* sp. NRS20 with starch as a carbon source accumulated 0.082 g/l of PHB, which was about 23.83% of DCW and higher than sucrose (11%) and glucose (12%) as carbon sources [61]. Similarly, *Haloarcula* sp. CEJ40-10 accumulated approximately 72.8% of its total DCW as PHA when cultivated with 1% (w/v) available starch as the carbon source, which was a significantly higher ratio per cell mass compared with other studies [62].

There have been increasing efforts to cultivate halophilic archaea using wastes for the sustainable production of PHA. *Hfx. mediterranei* could utilize olive mill wastewater as a sole carbon source, which was a highly polluting waste from the olive oil industry. The optimized medium containing 15% (v/v) of olive mill wastewater showed the highest PHA yield (0.2 g/l) and 43% content of its total DCW [63]. Using a macroalgal hydrolysate medium containing 25% Ulva sp. hydrolysate based on a rich medium for *Hfx. mediterranei*, PHA production reached 2.2 g/l, corresponding to 57.9% of dry cell weight (DCW) [64]. *Haloarcula* sp. CEJ40-10 achieved PHA production levels ranging from 0.88 to 1.2 g/l, corresponding to approximately 2% of the DCW, when cultivated on sugar factory wastewater at varying concentrations (0–40 g/l) as the sole carbon source, representing the first report of PHA production using sugar industry wastewater [62]. Using carboxylate-rich nutrients derived from food waste containing rice, ground beef, and raw cabbage, *Hfx. mediterranei* achieved PHBV production levels of 43–57% of cell dry weight (CDW), representing a 55% higher yield compared with glucose [65]. When agricultural wastes such as enzyme or acid-treated sugar beet, corn cob, and hazelnut husk were used as alternative carbon sources, *Haloarcula* sp. TG1 produced PHA of approximately 16.5–45.6% of DCW, with the highest productivity observed in enzyme-treated sugar beet as a carbon source [66]. PHA production was possible through the recycling of high-salt crude glycerol waste generated during the biodiesel production process by *Hfx. mediterranei*. By using crude glycerol diluted three times after removing long-chain fatty acids through acid treatment, the PHA yield was increased by 40% and the salt reduction effect required for cultivation was improved by 46% [67]. A 1:1 (w/w) mixed carbon source consisting of silkworm excrement extract and glucose significantly enhanced PHBV production by *Hfx. mediterranei*, reaching 1.73 g/l, which represents a 26% increase compared with fermentation conducted without silkworm excrement [68]. These results suggest that wastes from diverse industrial sectors can be utilized as alternative carbon sources, replacing conventional substrates and enabling higher PHA production than that achieved with standard culture media.

## PHA Overproduction and Monomer Ratio Control

**Fig. 3:**
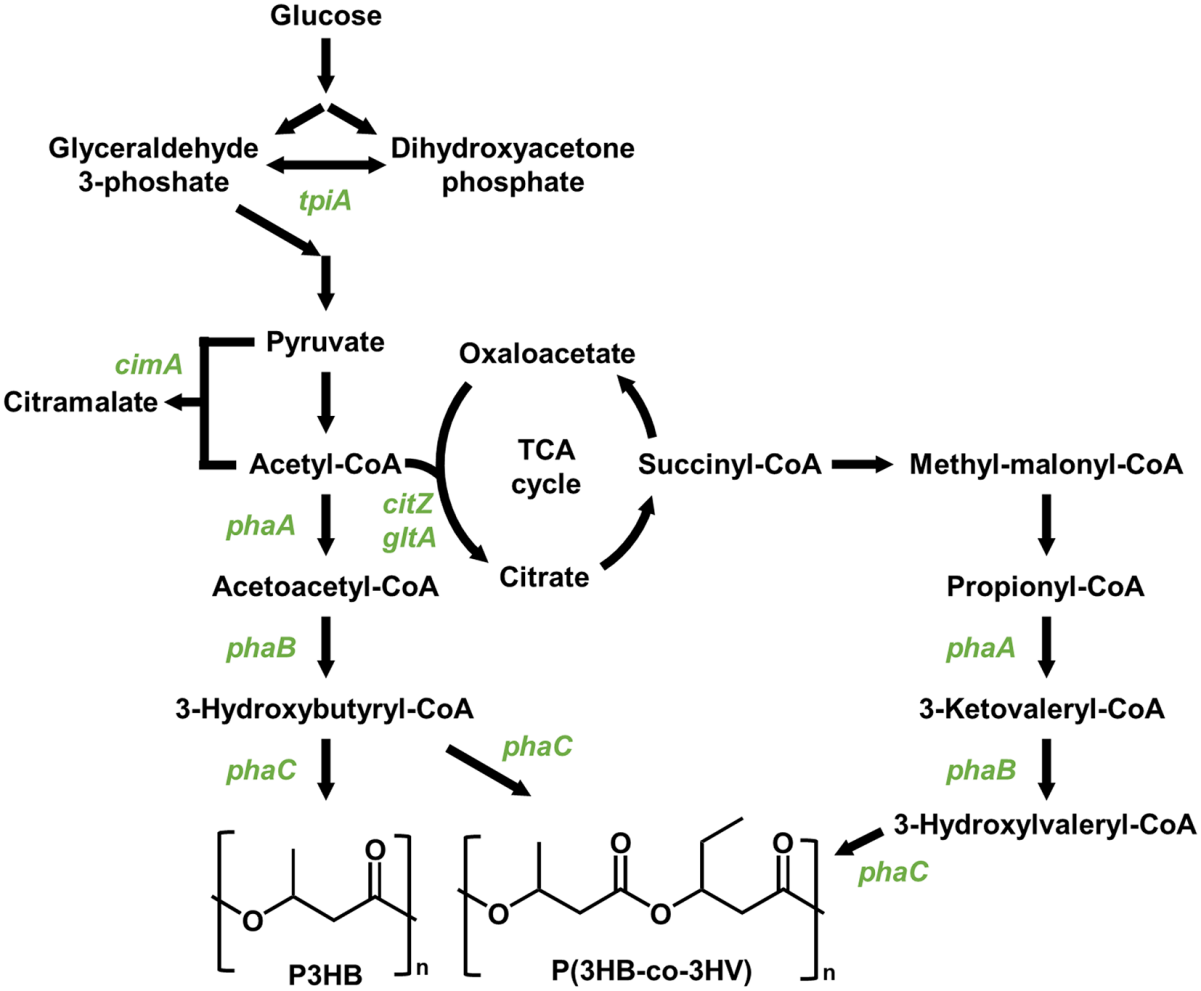
Biosynthesis pathways for polyhydroxyalkanoates production from halophilic archaea. Genes indicated in green color are involved in PHA overproduction and monomer ratio control. *tpiA*, triosephosphate isomerase; *cimA*, citramalate synthase; *citZ* and *gltA*, citrate synthase; *phaA*, 3-ketoacyl-CoA thiolase; *phaB*, 3-ketoacetyl CoA reductase; *phaC*, PHA polymerase or synthase.

Most recent studies have focused on halophilic bacteria such as *Halomonas* spp. [69-71]. To enhance PHA overproduction and control monomer composition, synthetic biology and metabolic engineering approaches have been applied to halophilic archaea (Fig. 3). Several reports describing only engineered haloarchaea for PHA production, these efforts have largely been limited to recombinant model strains of halophilic archaea, particularly *Hfx. mediterranei*. Genome-scale engineering of an EPS-deficient *Hfx. mediterranei* strain, including deletion of the phosphoenolpyruvate synthetase-like (*pps*-like) gene, increased PHBV production to 2.86 g/l (54.4% of DCW), accompanied by enhanced expression of the *pha* gene cluster (*phaR*–*phaP*–*phaE*–*phaC*) [72]. Furthermore, deletion of *phaC* relieved *pps*-like gene–mediated negative feedback on the alternative synthase *phaC1*, increasing the 3HV fraction in PHBV from 10.9% to 35.7%. These results provide mechanistic insight into the regulatory control of PHA biosynthesis in haloarchaea [72, 73]. CRISPRi-based repression of the citrate synthase genes *citZ* and *gltA* in the TCA cycle redirected carbon flux toward acetyl-CoA, a critical precursor for PHBV biosynthesis, increasing production by 76.4%, from 1.78 to 3.14 g/l [74]. Furthermore, genomic integration of the CRISPRi crRNA cassette, rather than plasmid-based expression, reduced metabolic burden. This strategy shortened the cultivation time from 5 to 3 days, and further enhanced PHBV production by 165%, reaching 4.13 g/l [74]. Improving the *pha* gene cluster located on the native pHM300 megaplasmid of *Hfx. mediterranei*, PHBV production was enhanced by controlling chromosome and plasmid copy numbers through deletion of replication origin genes [75]. Strain lacking *oriC1*, *oriC2*, and *oriC3* showed a 1.47–2.75-fold increase in *pha* gene expression due to elevated plasmid copy number. Despite reduced growth, PHBV production increased by 12.1% to 8.11 g/l, accompanied by a 21.2% increase in cell size, indicating improved intracellular PHBV accumulation [75].

Overexpression of endogenous genes unique to *Hfx. mediterranei* also positively influenced PHA production. Using a genome-scale metabolic model that accounted for carbon source utilization, triosephosphate isomerase (*tpiA*) was identified as a key gene supporting both cell growth and PHBV biosynthesis. Accordingly, replacing the native *tpiA* promoter with the stronger endogenous P_2422_ promoter increased PHBV production by 44%, from 1.50 to 2.16 g/l [76]. Similarly, replacement of the native promoters of (R)-citramalate synthase and β-ketothiolase in the genome of *Hfx. mediterranei* increased PHBV production by 35%, from 2.46 to 3.33 g/l, while the molar fraction of 3HV increased by 18.4%, a change directly associated with altered PHBV material properties [77]. A strategy involving the overexpression of genes directly associated with PHA biosynthesis has also been reported. An engineered *Hfx. mediterranei* strain, carrying plasmid-based copies of *phaE* and *phaC*, achieved 32.9% PHBV content, representing a 20% increase, when cultivated with 100 mM KNO_3_ as the nitrogen source. In addition, the proportion of 3-hydroxyvalerate (3HV) monomers in the PHBV copolymer increased by 40% [78]. Studies have also explored the introduction of novel monomers into PHBV through heterologous gene expression. In *Hfx. mediterranei* strain expressing 4-hydroxybutyrate (4HB)-CoA transferase/synthetase genes from *Nitrosopumilus maritimus*, production of the terpolymer P[(3HB)-co-(3HV)-co-(4HB)] was achieved instead of PHBV. Furthermore, supplementation with γ-butyrolactone increased the 4HB content in the terpolymer to 0.7 g/l, corresponding to 52 mol% of the 4HB fraction [79].

## Food and Pharmaceutical Functional Materials Biosynthesis and Its Properties

Most haloarchaea exhibit a bright red–orange coloration due to the accumulation of carotenoid pigments in their cell membranes. As a result, hypersaline environments approaching NaCl saturation often appear reddish in color, reflecting the high abundance of carotenoid-producing haloarchaea [80]. Carotenoids in archaeal cell membranes contribute to adaptation to hypersaline environments by acting as structural components that stabilize the membrane and facilitate the controlled diffusion of ions and oxygen molecules. In addition, haloarchaeal carotenoid pigments have also been reported to protect cells from UV radiation and to contribute to photoreactivation processes [81]. As a result, these carotenoids contribute to the stabilization of archaeal cells under extreme osmotic and oxidative stress conditions. Haloarchaeal carotenoids were first investigated in the 1960s. However, comparative studies between haloarchaeal carotenoids and those from other organisms remained limited until the latter half of the 20th century. In the 1970s, haloarchaea were first reported to produce major carotenoids belonging to the C50 family, predominantly bacterioruberin (BR) and its derivatives [82, 83]. Since then, research on haloarchaeal BR has expanded rapidly, with increasing focus on their biosynthesis, biological functions, and practical applications. BR has attracted significant interest as a functional ingredient for food, feed, and pharmaceutical applications due to its strong antioxidant activity and protective properties. Consequently, investigations into both haloarchaeal production systems and their functional properties have become increasingly important for developing scalable and sustainable sources of these valuable bio-compounds (Table 3).

## BR Biosynthesis Pathway in Haloarchaea

The first study elucidating the carotenoid biosynthetic gene pathway in haloarchaea was reported in 2015 [84]. In *Har. japonica*, mutants of these genes were constructed, and the carotenoid profiles of the resulting strains were analyzed. These studies demonstrated that *Har. japonica* encodes a carotenoid 3,4-desaturase (CrtD), a bifunctional lycopene elongase and 1,2-hydratase (LyeJ), and a C50 carotenoid 2'',3''-hydratase (CruF), respectively. Comparative genomic analysis of 11 halophilic archaeal species revealed that the *crtD*–*lyeJ*–*cruF* gene cluster is conserved, while the copy number of genes encoding the same enzymes varies among species [85]. All three genes exhibit high sequence conservation among species belonging to the same genus. While comparative analyses have revealed high amino acid sequence homology among BR biosynthetic genes across halophilic archaea from multiple genera, including *Halorubrum*, *Haloferax*, *Halobacterium*, and *Haloarcula* [86]. In addition to the core genes, phytoene synthase (CrtB) was also found to be clustered within the same gene cluster in certain species [85, 86]. Driven by these biosynthetic pathway studies, research on BR production in various halophilic archaea has steadily increased. Beyond the representative model organisms *Hbt. salinarum* and *Hfx. mediterranei*, BR production and the corresponding biosynthetic genes have been reported in numerous halophilic archaeal species [87-89]. Recently, BR production was achieved for the first time in a bacterial host through metabolic engineering using genetic elements derived from halophilic archaea, providing the functional validation of key BR biosynthetic genes [90].

## Culture Optimization for BR Production

Early studies on haloarchaeal carotenoid production focused on simple and one-factor-at-a-time (OFAT) optimizations. Adjusting individual culture conditions, such as temperature, pH, and salinity, can significantly improve bacterioruberin yields. The first study reached up to 0.604 A_494nm_/mL broth of carotenoid production by *Hfx. mediterranei* ATCC 33500 when 5% sodium chloride, 0.1% sodium acetate, and 8% magnesium sulfate are used as two-stage fermentation [91]. Based on the effect of the C/N ratio on BR production in *Hfx. mediterranei*, a two-stage fermentation strategy was applied to examine carotenoid accumulation. Cells were first grown in a nutrient-rich medium with a high C/N ratio and subsequently exposed to osmotic stress under carbon-limited conditions with a low C/N ratio, resulting in BR production of 58.49 mg/l, corresponding to 0.27% (w/w) of the total dry cell weight [92]. *Hfx. mediterranei* also grown in the presence of 2.5% (w/v) glucose or starch produced close to 100 mg/l of BR production [93]. These simple optimizations, though done OFAT, lay the groundwork by identifying favorable baseline conditions and substrates for each strain. To further enhance yields, several studies have employed statistical optimization via RSM. For example, *Hfx. marinum* MBLA0078 achieved 2.80 mg/l under OFAT and RSM-optimized fed-batch conditions in a 7 L fermenter, resulting from adjusting fish peptone, NaCl, KCl, and incubation time. The improvement corresponded to about a 12-fold increase in productivity [94]. Similarly, *Hrr. ruber* MBLA0099 was optimized by simultaneously optimizing factors like yeast extract concentration, pH, NaCl, inoculum size, and incubation time. In scale-up 7-L batch fermentation, *Hrr. ruber* achieved a maximal productivity of 0.492 mg/l/day, which was about a 6-fold improvement over the initial condition [95].

## Cost-Effective BR Production and Extraction

Beyond the above approaches, various other strategies have been explored to maximize bacterioruberin production according to the advantages of halophilic archaea. Using low-cost agro-industrial wastes such as starch residues from the candy industry, *Hfx. mediterranei* showed increased BR concentration reached 97.39 mg/l when the concentration of starch residues was 2.5% (w/v) [96]. A recent study optimized the open cultivation of *Halorubrum* sp. HRM-150 without strict sterilization, simplifying the process and improving cost-effectiveness for industrial BR production. Under these conditions, a 7 L open fermenter achieved approximately 7.3 mg/l of BR within 96 h [97]. Several studies have focused on maximizing bacterioruberin recovery to reduce overall processing costs. In *Halorubrum* sp. HRM-150, C50 carotenoids were efficiently extracted by suspending and homogenizing cell pellets in methanol (1:30, w/v) for 10 min, followed by incubation at 30°C for 30 min in the dark and subsequent centrifugation and vacuum rotary evaporation at 30°C. This procedure resulted in a 1.84-fold increase in carotenoid recovery [98]. In addition, the use of bio-based solvents has been explored as a sustainable alternative. A 150 mM aqueous gamma-valerolactone solution, applied at a solid–liquid ratio of 0.15 and pH 7, achieved a maximum carotenoid yield of 968 μg/g biomass after 95 min of extraction from *Hbt. salinarum* R1 [99]. These strategies contribute to progressively improving the productivity and economic viability of haloarchaeal BR production.

## BR Application for the Food Industry

Recent studies have explored the biotechnological potential of BR, focusing on its value not only as a pigment but also for its strong antioxidant activity (Table 4). In the food industry, a bacterioruberin-rich carotenoid extracted from *Hrr. ezzemoulense* DSM 19316 provided effective protection when added to fish oil. Carotenoid extracts at 500 ppm effectively delayed oxidative degradation, maintained fatty acid profiles, and reduced the levels of volatile oxidation products, which serve as primary indicators of lipid oxidation [100]. Similarly, when added to emulsified sausage at 50 mg/kg, the bacterioruberin-containing extract demonstrated potential for food preservation by improving color stability and reducing peroxide value, free fatty acid content, and thiobarbituric acid reactive substances associated with lipid oxidation [101]. In addition, BR has strong potential as a functional food additive due to its distinctive red color, suitability as a natural food colorant, and biological activities, particularly its antioxidant properties [102].

## BR Application for the Pharmaceutical Industry

In line with its antioxidant activity, numerous studies have reported positive pharmaceutical properties of BR, representing various pathological conditions. Human cells can benefit from carotenoids as antioxidant agents through their ability to scavenge free radicals or to activate endogenous antioxidant defense pathways. For example, treatment with 2 mg/l of a carotenoid extract from *Halovenus aranensis* increased both mRNA and protein levels of nuclear factor erythroid related factor 2 (NRF2), indicating activation of the NRF2-Kelch-like ECH-associated protein 1 (KEAP1)-dependent antioxidant defense signaling pathway [103]. Pretreatment of lipopolysaccharide (LPS)-stimulated macrophages with *Haloarcula* sp. OS BR extract resulted in upregulation of NRF2 and decreased levels of the pro-inflammatory cytokines TNF-α and IL-6, and its downstream target gene heme oxygenase-1 (HO-1) [104]. Similarly, in a muscle cell model C2C12 myotube treated with LPS, the mRNA expression of TNF-α, IL-6, inducible NO synthase (iNOS), and cyclooxygenase-2 (COX-2) was significantly attenuated by 25–100 μg/ml of BR extracts from *Hfx. marinum* [105]. Two studies have also unveiled the *in vitro* anticancer potential of BR from haloarchaea. BR extracted from *Har. hispanica* A15 induced apoptosis in approximately 50% of MCF-7 breast cancer cells and cell cycle in S and G2 phases. In addition, BR-treated MCF-7 cells significantly upregulated the expression of apoptosis-related markers, including caspase-3, Bax, and caspase-8, indicating activation of proteolytic apoptotic signaling pathways leading to cell death [106]. BR extracts from *Hfx. mediterranei* treatment induced pronounced apoptotic cell death in K562 and THP1 myeloid leukemia cells, reducing cell viability by approximately 50% at 18.50 and 5.03 μg/ml at 72 h, respectively. BR extracts significantly inhibited cell proliferation and induced cell cycle arrest at the G2/M phase, accompanied by a reduction in S-phase or G0/G1 populations, triggering a concentration-dependent increase in intracellular ROS levels [107].

*In vivo* studies evaluating the biological activity of BR have also been reported. When *Caenorhabditis elegans* were fed 3 μM of BR derived from *Hrr. rubrum* and subsequently exposed to 2 mM H_2_O_2_ for 5 h, the survival rate of the nematodes was approximately threefold higher than that of untreated controls [95]. *In vivo* high-fat diet (HFD) mouse model demonstrated that fed 250–500 mg/kg BR from *Hbt. salinarum* significantly increased the activities of endogenous antioxidant enzymes and markedly reduced lipid peroxidation levels. BR supplementation significantly suppressed body weight gain induced by the HFD and improved serum lipid profiles in a dose-dependent manner, including reductions in total cholesterol, triglycerides, low-density lipoprotein-cholesterol, and the atherogenic index, while restoring high-density lipoprotein-cholesterol levels [108].

## BR Application with Drug Delivery System

Owing to these beneficial effects, there is a growing interest in utilizing BR as a therapeutic agent by incorporating it into drug delivery systems. Accordingly, multiple nanoparticle-based strategies have harnessed BR for therapeutic benefit in varied disease models. For example, co-encapsulation of *Hrr. tebenquichense* BR extracts with the corticosteroid dexamethasone (Dex) in ultra-small archaeal lipid nanoparticles were investigated as an intestinal repair therapy. BR/Dex nanoparticles targeted gut macrophages and markedly attenuated inflammation in an LPS-stimulated Caco-2/THP-1 co-culture, reducing TNF-α and IL-8 levels by 65% and 55%, respectively. These also decreased oxidative stress by approximately 60%, and restoring epithelial barrier function in an *in vitro* colitis model [109]. In the same research group, an *in vitro* skin inflammation model, both THP-1 macrophages and 3T3 fibroblasts-targeted nanoparticle co-loaded with vitamin D3 and 352 μg/mL of BR from *Hrr. tebenquichense* dramatically suppressed IL-6 and IL-8 and promoted wound closure [110]. Similarly, nebulizable archaeal nanovesicles incorporating BR from *Hrr. tebenquichense* exhibited potent anticancer effects in lung carcinoma cells. A BR concentration of 0.15 μg/mL achieved 50% growth inhibition comparable to cisplatin, yet at doses non-toxic to THP-1 macrophages. This inhalable formulation also reprogrammed tumor-associated macrophages toward a pro-inflammatory phenotype, as evidenced by decreased CD204 (M2 marker) and increased IL-6 and TNF-α production [111]. Another study found that encapsulating BR from *Hrr. tebenquichense* in archaeosomes, which contained 9.6 μg BR/mg lipids, versus conventional liposomes improved its stability and bioactivity. The BR-loaded archaeosomes protected red blood cells from free radical-induced hemolysis and were efficiently internalized by macrophages, imparting TNF-α and antioxidant effects in LPS-induced macrophages comparable to those of BR-liposomes [112].

## Conclusion and Perspectives

Haloarchaea have emerged as promising microbial platforms for biomanufacturing due to their exceptional tolerance to extreme salinity, unique metabolic features, and ability to produce structurally and functionally distinctive bio-compounds. Significant improvements in target compounds productivity have been achieved through culture optimization, utilization of low-cost and waste-derived substrates, open fermentation systems, and genetic engineering approaches. Haloarchaeal enzymes are particularly attractive because of their distinctive physicochemical properties, stability under harsh conditions, and broad application potential. In addition, haloarchaea have served as a key model organism for advancing PHA production and elucidating regulatory mechanisms that control polymer yield and monomer composition. Growing interest in the C50 carotenoid bacterioruberin has further highlighted its strong antioxidant, anti-inflammatory, and therapeutic properties. Despite this progress, several practical challenges remain. Industrial-scale cultivation of haloarchaea is hindered by the difficulty of achieving high-cell-density fermentation under hypersaline conditions, which can cause corrosion of bioreactor materials, increased viscosity, and high energy demand for downstream processing. Cultivating generally aerobic halophilic archaea is difficult because high salt concentrations markedly decrease dissolved oxygen levels in the culture medium, and maintaining sufficient oxygen transfer becomes even more problematic during large-scale fermentation processes. Moreover, while haloarchaeal enzymes are valuable, heterologous expression in conventional hosts is often pursued to improve production efficiency; however, successful expression can be limited by the requirement for high intracellular salt concentrations to maintain proper protein folding and activity. Comparative evaluations suggest that although heterologous systems offer scalability and cost advantages, native haloarchaeal hosts expect to remain superior for producing fully functional halophilic enzymes. Continued interdisciplinary research will be essential to fully unlock the biotechnological potential of haloarchaea. Overall, haloarchaea offer an attractive route to high-value bio-compounds even under extreme environmental conditions.

## Figures and Tables

**Table 1:** Recent advances in the production and properties of cellulase, protease and amylase from haloarchaea.

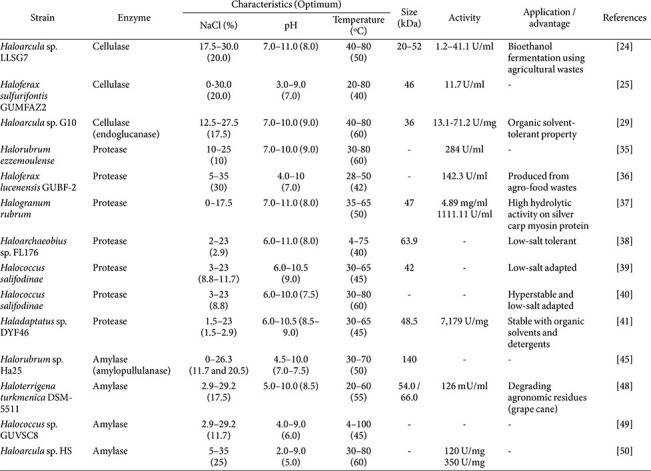

**Table 2:** Recent advances in the production of polyhydroxyalkanoates from haloarchaea.

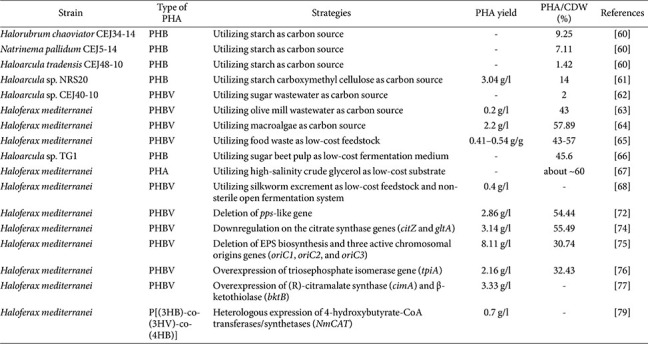

**Table 3:** Recent advances in the production and extraction of bacterioruberin from haloarchaea.

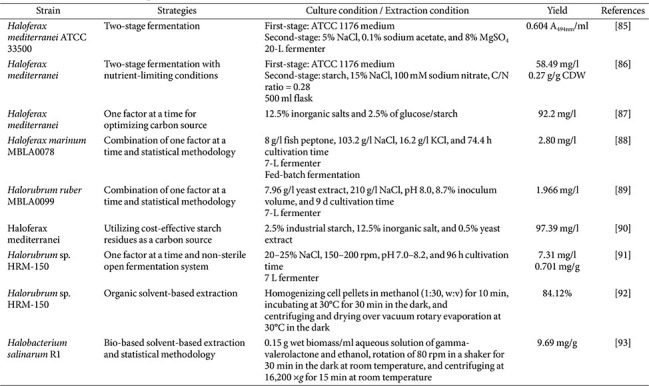

**Table 4:** Recent advances in the functional properties of bacterioruberin from haloarchaea.